# A Complex of Lance Flies (Diptera: Lonchaeidae) Infesting Figs in Veracruz, Mexico, with the Description of a New Species †

**DOI:** 10.3390/insects16050458

**Published:** 2025-04-27

**Authors:** Rodrigo Lasa, Laura Navarro-de-la-Fuente, Iain MacGowan, Trevor Williams

**Affiliations:** 1 Instituto de Ecología AC, Xalapa 91073, Veracruz, Mexico; 2 Facultad de Biología, Universidad Veracruzana, Zona Universitaria, Xalapa 91090, Veracruz, Mexico; mlnavarrodelafuente@yahoo.com.ar; 3 National Museum of Scotland, Edinburgh EH5 1JA, UK; imacgowan9@gmail.com

**Keywords:** *Silba adipata*, *Neosilba* spp., *Ficus carica*, oviposition, COI gene sequence, phylogenetic analysis

## Abstract

The black fig fly, *Silba adipata*, and a complex of *Neosilba* species have emerged as major fig pests in Veracruz, Mexico. To assess infestation patterns and species composition, we sampled figs during one production cycle (March–October 2024) in two plantations. A total of 1647 *S. adipata* (821 females, 826 males) and 1250 *Neosilba* spp. (628 females, 622 males) emerged from samples. One species, *Neosilba recurva*, is described as new based on the characteristics of the male genitalia and COI sequencing. *Neosilba* spp. outnumbered *S. adipata* in the preharvest period, after which *S. adipata* became dominant. *S. adipata* emerged ~14 days earlier than *Neosilba* spp. Infestation by *S. adipata* caused uniform fig reddening, while *Neosilba* spp. produced patchy redness near larval exit holes. Approximately 65% of figs were lost to lonchaeid infestation preharvest. Based on male genitalia, we identified *Neosilba batesi* and *N. recurva* sp. nov. (that together comprised 64% of samples), as well as *Neosilba* sp.1 (26%) and *N. glaberrima* (10%), but COI sequences revealed greater diversity. *Neosilba batesi* formed a clade that did not match a Floridan sequence. *Neosilba glaberrima* clustered near *N. pendula* from Brazil, but another sequence diverged, suggesting the presence of an additional, undescribed species, *Neosilba* sp.2? *Neosilba recurva* and *Neosilba* sp.1, together with the newly described *N. flavitarsis* from Veracruz, formed distinct genetic branches, supporting their specific status.

## Simple Summary

In recent years, fig production in Veracruz State, Mexico, has been severely affected by infestations of lance flies, the larvae of which develop within figs, making them unmarketable. The resulting losses can exceed 50% of the crop. We assessed the fig infestation patterns over one production cycle (2024) in two affected plantations. Infestation by *Neosilba* species and *Silba adipata* differed in the pre-harvest and post-harvest periods. *Silba adipata* emerged from infested figs approximately two weeks before *Neosilba* species. Figs infested by each genus of flies also differed in the signs of infestation (red coloration) and the presence of larval exit holes. By examination of male genitalia, *Neosilba batesi* and *N. glaberrima* were identified in addition to a new species named *Neosilba recurva* sp. nov. Analysis of COI gene sequences revealed significant diversity, indicating the presence of two additional species. This study highlights the need for expanded molecular research on the Lonchaeidae family.

## 1. Introduction

The fig, *Ficus carica* L. (Moraceae), is an ancient crop from the Mediterranean and south-west Asia [1]. Mexico is not a major fig producer, but production is increasing and currently occupies an area of 1843 Ha, with an annual production of 12,500 tons, mainly in the states of Morelos, Hidalgo, Puebla, and Veracruz [2].

Fig production in central Mexico involves harvesting figs for immediate consumption or exportation. In Veracruz State, green figs are harvested before maturity and are processed into candied dry figs or conserved in syrup. The center of fig production in Veracruz is located in the municipality of Tatatila, a region with a temperate-humid climate. Figs are harvested during two main periods of the year: a small-scale harvest in March–April and a larger main harvest during the summer in July–August.

Fig growers in Veracruz do not usually apply any phytosanitary treatments, as no significant pests had been observed until 2023, when several growers reported high infestations of lance flies (Diptera: Lonchaeidae), including the black fig fly, *Silba adipata* McAlpine, 1956. This is a monophagous pest that lays eggs in small clusters beneath the scales of the ostiole of unripe figs [3]. The larvae emerge and feed on the immature receptacle tissue, damaging the fig internally and causing premature fig drop. On reaching maturity, the larvae exit the fig by cutting a hole in the cuticle and fall to the ground to pupate. This pest originates from the Mediterranean and Middle East regions but was first detected in Morelos State, Mexico in 2020 [4]. By 2021, its presence had been recorded in several other states in central Mexico [5,6] and California, USA [7]. By 2023, fig growers in Veracruz state were reporting losses that exceeded 50% [8].

Preliminary sampling in October 2023 using torula yeast baited traps revealed the presence of *S. adipata* and several species of the genus *Neosilba* Waddill and Weems, 1978. This lonchaeid genus is native to the Neotropics, including southern Florida and the islands of the Caribbean [9]. Most *Neosilba* species are considered to be secondary invaders of fruits that were initially attacked by tephritid fruit flies (Diptera, Tephritidae), or other primary fruit pests [10,11,12]. However, some species of *Neosilba* are likely to be primary invaders of different fruits within the families Myrtaceae, Rosaceae, Rubiaceae, Moraceae, Anarcadiaceae, Annonaceae, and Sapotaceae [13,14]. At least five species of *Neosilba* have been associated with figs in Brazil [12], and recently, a new species, *N. flavitarsis* MacGowan and Lasa, 2025, was reared from figs collected in Veracruz, Mexico in 2023 [15].

Given the rapid appearance and uncertain identity of fig-infesting lonchaeid species in Tatatila, Veracruz, this study aimed to sample figs which became infested throughout the 2024 production cycle. During this period, adult lonchaeids that emerged from figs were collected, counted, and identified. Emergence was also observed from figs displaying different signs of infestation. Due to the complex taxonomy of this family and the paucity of genetic information, a selection of fig-infesting adults was subjected to phylogenetic analysis, with unexpected results. These findings have identified potential new lines of research on the classification, multi-species interactions, and control strategies for these rapidly emerging lonchaeid pests.

## 2. Materials and Methods

### 2.1. Monitoring of Fig Infestation

Sampling was performed in two fig plantations located <1 km from the village of Tenexpanoya in the municipality of Tatatila, Veracruz State, Mexico. Site 1 comprised a planted area of 1500 m^2^ (19°39′32.02″ N; 97°8′36.81″ W, 1840 m elevation), whilst site 2 comprised a planted area of 2500 m^2^ (19°39′38.44″ N; 97°8′35.71″ W, 1825 m elevation). This region has a temperate climate with an average temperature of 20 °C and an average annual precipitation of 1346 mm that mainly falls between June and October, with a dry season from November to May [16]. This region cultivates a fig that has been propagated in the area for many decades and is presumed to have originated from the “Brown Turkey” variety. At both sites, fig plants were 2.5–3.0 m in height, planted at intervals of 2 m, and had 4–6 main branches. No phytosanitary measures or fertilizer treatments were applied to the crops in 2023 or during our sampling in 2024.

Based on the stages of fig infestation by *S. adipata* in the Mediterranean region [17], sampling began on 13 March 2024 and continued on a weekly basis until 11 July, when it was interrupted due to the harvesting of figs. Sampling restarted on 22 August but at intervals of 7–18 days due to the low quantity of figs available. The final sample was taken on 15 October 2024. At each sampling event, infested figs, usually in a phenological stage between 71 and 75 on the BBCH scale [18], were identified by their visual appearance and collected in 16 × 30 × 12 cm plastic trays, covered with an anti-insect mesh. These were transported to the laboratory for processing. The quantity of the collected figs varied depending on the number of infested figs available in the orchard at each sample time. The sampling effort involved one person collecting figs for approximately 20 min at each site on each date.

Each sample was weighed upon arrival at the laboratory and then returned to a plastic tray placed inside a nylon mesh cage (90 × 60 × 90 cm). A 5 mm layer of vermiculite was placed over a layer of paper towels in the bottom of each tray to maintain humidity and prevent excess liquid collecting, which may have caused the figs to rot. Samples that exceeded 1 kg were divided into additional trays so that none exceeded 0.8 kg of figs per tray. From the date of collection, the number of emerged adult lonchaeid flies was recorded daily for a period of 45 days. Adult flies were placed in glass vials, euthanized by freezing (−20 °C), and preserved in 70% ethanol for subsequent identification (Section 2.4).

### 2.2. Emergence of Flies from Figs

Figs that showed different signs of infestation were examined before and after the fig harvest in August 2024. During the main fig growing period (16 April–4 July 2024), a sample of 40 figs with different signs of infestation was collected. Figs were individually weighed and their equatorial diameter measured at the widest point. Figs were then placed individually in plastic cups (470 mL capacity) and covered with nylon mesh. Each cup contained a 5 mm deep layer of vermiculite as a substrate for pupation. The vermiculite was moistened at two-day intervals with 3% (*wt*/*vol*) sodium benzoate solution to maintain the humidity and suppress the growth of fungi. The emergence of adult flies at 25 ± 1 °C was recorded daily over a period of 45 days; the flies collected from each cup were examined, sorted by sex, and identified.

The number of holes made by larvae as they exited the fig to pupate in the soil [17] was counted in a sample of 130 randomly selected figs. The position of these holes was classified into three groups: (i) on the lower third of the fig including the ostiole, (ii) on the central third of the fig, and (iii) on the upper third of the fig attached to the pedunculus.

After the harvest, another sample of 40 figs without the presence of an exit hole was collected at the end of August 2024 and placed in individual plastic cups as described above. Figs with holes were almost completely absent in the orchards and were not evaluated in the post-harvest period. The emergence of adult flies from each cup was recorded daily for 45 days, and the adult lonchaeids were collected, sorted by sex, and identified.

### 2.3. Fig Production and Prevalence of Infestation

The total number of figs was estimated on six trees that were randomly selected at each site on each week. These counts were performed from 6 June until the beginning of the harvest on 11 July 2024. Each tree was divided in half and all figs on one half of the tree were counted, regardless of the size of the figs. The number of figs that had the characteristic signs of infestation was noted. Similarly, following the harvest, the number of figs remaining on each tree and the prevalence of infestation were also determined on ten randomly selected trees at each site.

### 2.4. Species Identification and Phylogenic Analysis

Adult flies were examined under a stereomicroscope to determine species and sex. The distinction between *S. adipata* and other *Neosilba* species was made using taxonomic keys [9,19,20,21,22]. *Neosilba* was primarily differentiated from *Silba* by the presence of a group of long dark setae in the otherwise pale fringe of the calypter. The identification of males was based on the characteristics of the genitalia; no taxonomic keys are available for *Neosilba* females.

For detailed examination of the genitalia, adult specimens were micro-pinned from ethanol and staged with accompanying data labels. The genitalia were dissected from the abdomen, cleared in 10% KOH before examination, and subsequently stored in a glycerol filled micro-vial attached to the specimen pin. The taxonomic terminology used in describing the new species follows that of MacGowan and Rotheray [23].

#### 2.4.1. COI Gene Sequence Analysis

One female and one male of *S. adipata* and 15 male *Neosilba* spp. were subjected to Cytochrome oxidase subunit I (COI) gene sequence analysis in an attempt to clarify the relationships among these individuals. For this, DNA extraction from the adult flies was performed using the DNeasy Blood & Tissue Kit (Qiagen, Hilden, Germany), as described previously [15]. PCR amplification of the mitochondrial COI gene was performed using primers C1-J-1718 and C1-N-2191 [24] on a SureCycler 8800 thermocycler (Agilent Technologies, Santa Clara, CA, USA) under the following conditions: initial denaturation at 95 °C for 1 min, followed by 40 cycles at 95 °C for 15 s, 50 °C for 30 s, 72 °C for 45 s, followed by a final extension step at 72 °C for 5 min. The amplicons (525 pb) were purified using the Wizard SV Gel and PCR System Clean-Up kit (Promega, Madison, WI, USA) and sent to Labsergen Langebio (Irapuato, Mexico) for Sanger sequencing. The resulting sequences were assembled and edited using BioEdit v.7.0.5 software [25] and were compared to nucleotide sequences in GenBank using BLAST search software v.2.16.0 [26,27].

#### 2.4.2. Phylogenetic Analysis

For the phylogenetic analysis, the COI gene sequences obtained in this study and those of the related species were aligned using the MAFFT v7 online service [28] and Mega MEGA 7 software [29]. The best nucleotide substitution model was estimated using the Akaike Information Criterion (AIC) within jModelTest 2 [30]. A phylogenetic tree was generated using the maximum likelihood (ML) method in RAxMLGUI 2.0 with the GTR+G evolutionary model [31]. Bootstrap analysis was performed on 1000 repetitions. In addition, a phylogenetic analysis was performed using Bayesian inference with the MrBayes v 3.2.6 software [32] for 5 million generations. The resulting phylogenetic tree was visualized and edited using FigTree v1.4.3 [33]. Numbers at branch nodes indicate bootstrap values (BS ≥ 70) and Bayesian Posterior Probabilities (BPP ≥ 0.9).

### 2.5. Statistical Analyses

The median time elapsed for adults to emerge from field-collected figs was determined by Kaplan-Meier analysis and log-rank test. The mean weights and mean diameters of figs were compared by *t*-test or Welch’s *t*-test in the case of unequal variances. The frequencies of putative larval emergence holes per fig and their position on infested figs were compared by χ^2^ test. The mean numbers of figs per tree during different weeks of the fig production cycle were compared by fitting a generalized linear model (GLM) with quasi-Poisson distribution specified to account for overdispersion. All analyses were performed using the R-based package Jamovi, version 2.3.28 [34].

## 3. Results

### 3.1. Systematics

Genus *Neosilba* Waddill and Weems, 1978.

***Neosilba recurva*** MacGowan and Lasa sp. nov.

#### 3.1.1. Description

*Holotype Male*: *Head*: Eye bare; frons sub-shining black, ratio of frons width at narrowest point above the lunule to eye width 1:2.1; frontal and interfrontal setulae short, approximately 0.2× length of orbital seta, longer setulae present on the anterior margin; orbital plate shining black, bare apart from the orbital seta; lunule black, parafacial and face sub-shining black; antenna black, postpedicel length to depth ratio 3.2:1; arista light brown basally, short plumose, plumosity at maximum extent 0.7× depth of postpedicel; anterior genal setulae in a single row of 5–7 along the mouth margin, noticeably stronger than the other setulae on the gena.

*Thorax*: Scutum sub-shining blue-black, covered in rather dense black setula approximately 0.3× as long as the orbital seta; anepisternum anteriorly with a vertical row of four setae, posteriorly with a vertical row of five on right, four on left, remainder of sclerite covered in long setulae, approximately 0.5× as long as the posterior setae; katepisternum with two setae located near dorsal margin, anterior slightly positioned slightly more ventral than posterior, a scattering of setulae located anterior to these on the anterior part of the sclerite; proepisternum and proepimeron each with a single seta; prosternum bare; scutellum; black scutum, slightly dulled by dusting, margin between lateral and apical setae with four long setulae on right side, setulae apart from one missing on left, two setulae between apical setae; yellowish-white calypter with a slightly darker margin, with a yellowish fringe within which, centrally placed, are approximately ten long black setulae 2.5× longer than the other setulae in the fringe; wing shows clear, veins brownish-black, wing length 4.8 mm; legs entirely black. halter black with a greyish stem.

*Abdomen*: sternite 1 bare, tergite 5 apically with marginal setulae.

**Figure 1:**
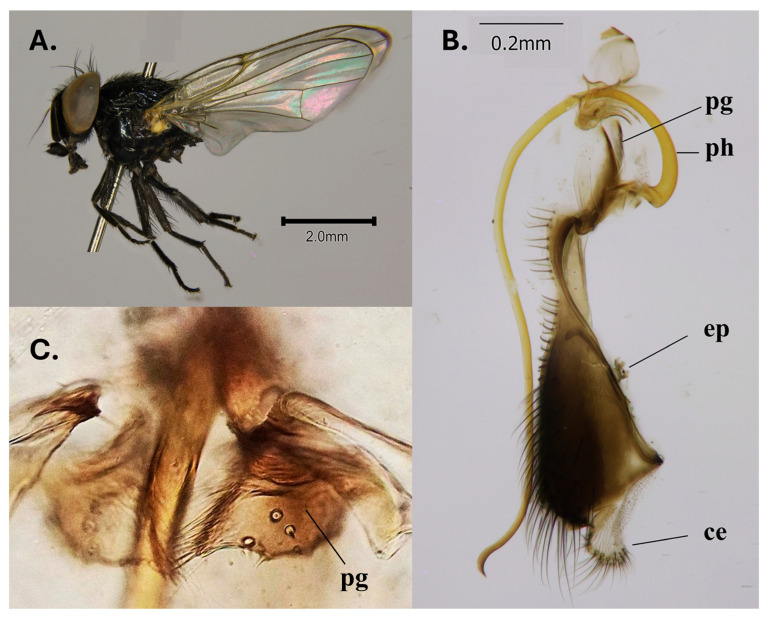
*Neosilba recurva* sp. nov. (**A**). Lateral view of holotype adult male with abdomen removed for dissection (for a view of a paratype see Supplemental Figure S1). (**B**). Male genitalia lateral view with 0.2 mm scale bar, pg—postgonite, ph—phallus, ep—epandrium, ce—cercus. (**C**). Detailed view of postgonite (pg), ventral view. A detailed description of adult dipteran morphology is given elsewhere [37] and with specific reference to Lonchaeidae [23].

*Male terminalia*: (Figure 1A–C) In lateral view, epandrium 2.3× as high as it is wide, greatly attenuated in the anterior third, bearing numerous strong setae along ventral margin and posteroventraly; cercus comprised a relatively small, rounded structure approximately 0.27× the height of the epandrium with numerous setae along the ventral and posterior margins; surstylus long and narrow, extending the full length of the epandrium, a row of regularly spaced strong setulae along entire ventral margin, with an inner surface with a uniform close-set row of eight black prensisetae along the posterior margin near the base of the cerci and three well-spaced black spicules forming a continuation of this row anteriorly; postgonite (Figure 1C) almost square in shape, its height slightly less than the basal width of the postgonite median margin, bearing a dense row of fine setulae, four to five small setae on surface; phallus, a simple curving tube of uniform thickness reaching posteriorly to extend beyond the posterior margin of the epandrium almost to the apex of the cerci, without processes or spicules, basal section semi-circular, basal plate with a dorsal-ventral orientation, apex strongly recurved.

*Female*: unknown.

Holotype: ♂, MEXICO: Veracruz, Tatatila, 19.6908 N, 97.1105 W, emerged from *Ficus carica* (L.), syconia, April–June 2024, leg. Rodrigo Lasa. Specimen deposited in National Museum of Scotland, Edinburgh, specimen code NMS-10030222.

Paratypes: with the same data as the holotype deposited in IEXA, Entomological collection of the Instituto de Ecología AC, Xalapa, Veracruz, Mexico.

Etymology: The specific epithet refers to the recurved apex of the phallus.

#### 3.1.2. Taxonomic Diagnosis

For our diagnosis, we ran the characters of *Neosilba recurva* through the most up to date key for *Neosilba* species [35]. This species was characterized as follows: a combination of male terminalia not hemispherical in shape, epandrium wider than the surstylus or at least 1/3 wider than long, phallus width similar throughout and with apex S-shaped; keys out as belonging to the *Neosilba pendula* (Bezzi, 1919) species-group. Within that species-group with the combination of the basal part of the phallus that is not circular in shape, without spicules, of almost of uniform thickness throughout its length and not swollen apically; this leads to the final couplet in the key which contains *Neosilba perezi* (Romero and Ruppel, 1973) and *Neosilba angusta* Galeano-Olaya and Canal, 2012. Nonetheless, *N. recurva* is clearly distinguishable from these two species.

In the original description of *Neosilba perezi*, it is stated that the posterior margin of surstylus bears a comb-like row of about 13–15 equal-sized black teeth (prensisetae), compared to the eight in *Neosilba recurva*. The phallus of *N. perezi* is described as rod-shaped, as long as the surstylus [36]. There is no mention in the text of a recurved apex, and the lateral figure of the genitalia provided ([36], p. 167: Figure 1b) does not indicate this. The lectotype of *N. perezi* was designated and described by McAlpine and Steyskall [20], who also illustrated the male genitalia (p. 135: Figures 41 and 42). They noted that the apex of the phallus is slightly enlarged in ventral and lateral views, and in the illustrations provided, the apex of the phallus is only slightly sinuate.

In addition to the description, photographs of the male genitalia of *Neosilba angusta* were provided by Galeano-Olaya and Canal [35] in their Figure 8. *Neosilba recurva* is distinguished from *N. angusta*, which has the surstylus with nine prensisetae on each side, two of which are located medially and separated from the row of the other seven. In *N. recurva*, there is no such gap in the row of prensisetae. The phallus, although long and thin in *N. angusta*, is not as sinuous as in *N. recurva*, and the basal plate has a posterior–anterior orientation. The postgonite of *N. angusta* is rather long and triangular in shape when compared to the rather square shape in *N. recurva*.

### 3.2. Monitoring Fig Infestation During the Growing Period

Systematic weekly sampling began on 13 March 2024, although the first infested figs were not collected until 19 April. From that date, both *S. adipata* and *Neosilba* spp. adults emerged from figs collected up to the end of the experiment on 15 October 2024 (Table 1). A total of 1647 *S. adipata* adults (821 females, 826 males) and 1250 *Neosilba* spp. adults (628 females, 622 males) emerged from a total of 22 kg of figs (approximately 65–80 figs/kg) collected from April to October (mean ± SE sample weight: 1.44 ± 0.28 kg).

The average number of *S. adipata* that emerged from each kilogram of figs increased gradually between April and early June, with an initial peak of infestation in figs collected on 13 June, followed by a larger high peak in figs collected on 4 July, immediately before fig harvest (Table 1). In contrast, the emergence of *Neosilba* spp. adults was fairly stable in figs collected between 3 May and 13 June but decreased notably in samples collected after that date. Individuals of *Neosilba* spp. outnumbered those of *S. adipata* in the preharvest period up to 20 June 2024, whereas after that *S. adipata* became the dominant species in field-collected figs (Table 1). Following the harvest, the emergence of both species remained close to pre-harvest levels, although the weight of figs sampled was generally reduced due to lower availability in the field.

The median (95% CI) time elapsed between fig collection and adult emergence from trays in the laboratory (25 ± 1 °C) was 17.5 days (17.0–21.5 days) for *S. adipata*, a significantly shorter time than the 30.5 days (29.7–33.4 days) registered for *Neosilba* spp. (Log-rank test, *p* < 0.001). The median elapsed time of emergence was similar for males and females of *S. adipata* (Log-rank test, *p* = 0.70) and for both sexes of *Neosilba* spp. (Log-rank test, *p* = 0.84) (Supplemental Table S1).

### 3.3. Signs of Fig Infestation

**Figure 2:**
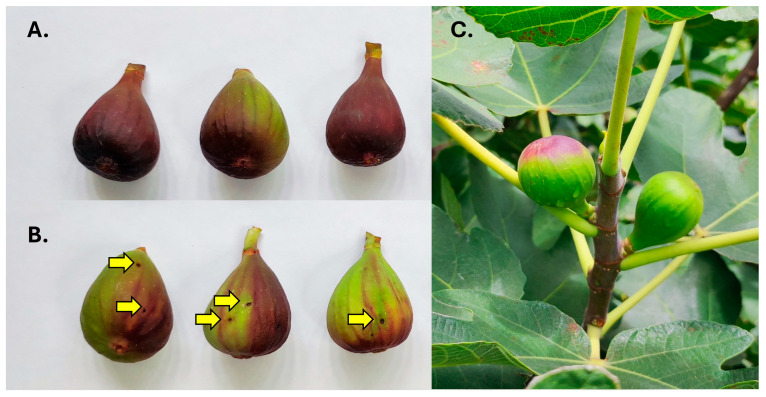
Two different patterns of infested fig detected in fig orchards; (**A**) figs that were infested by *Silba adipata* alone, and (**B**) figs with exit holes (yellow arrows) and patchy coloration that were mainly infested by *Neosilba* spp. (**C**) Infested figs (on left) were readily distinguishable in the field by their reddish coloration compared to uninfested green figs (on right).

Figs differed in their signs of infestation. Two patterns were detected that involved the presence of exit holes and differences in the coloration of infested figs (Figure 2A–C). Type A was characterized by a homogeneous premature red coloration that was particularly evident and intense around the ostiole, gradually fading towards the pedunculus (Figure 2A). These figs had no signs of exit holes and were only infested by *S. adipata*, with no emergence of *Neosilba* spp.

In contrast, Type B figs had one or two obvious holes (or occasionally three or four holes per fig) and were characterized by a mainly green appearance with patches of a diffuse premature reddening. The red coloration did not initiate close to or around the ostiole (Figure 2B). Infested figs were therefore clearly distinguishable from uninfested figs in the field (Figure 2C).

**Figure 3:**
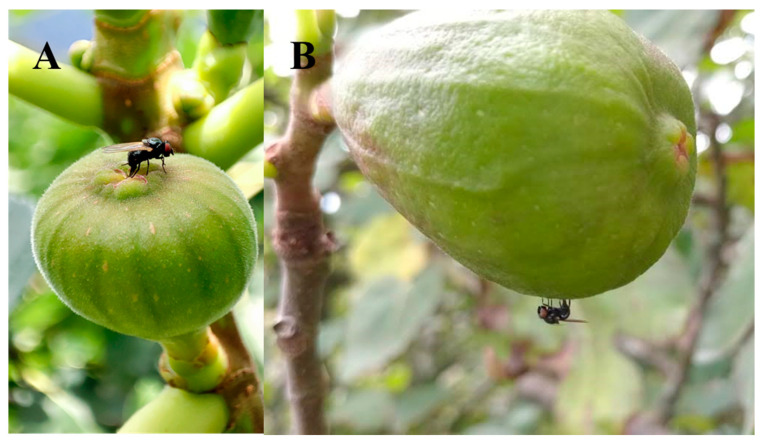
Oviposition behavior of (**A**) *Silba adipata* and (**B**) *Neosilba* spp. on figs in Veracruz, Mexico.

We initially believed that *Neosilba* spp. were infesting figs that had previously been infested by *S. adipata* and had used the larval exit holes made by *S. adipata* for their oviposition, which would be consistent with the behavior of a secondary invader. However, no *Neosilba* spp. females were observed in the field ovipositing in the larval exit holes. In fact, field observations revealed distinct behaviors—several females of *S. adipata* were observed ovipositing inside the ostiole (Figure 3A), whereas two *Neosilba* spp. females were observed attempting to oviposit around the equatorial region of the fig (Figure 3B), although we do not know whether oviposition by *Neosilba* spp. in the absence of larval exit holes was successful.

### 3.4. Emergence of Flies from Figs

#### 3.4.1. Samples Collected Prior to Harvest

Figs without an exit hole had a smaller mean diameter (t = 4.07, df = 78, *p* < 0.001) and a lower mean weight (t = 5.73, df = 78, *p* < 0.001) than figs with a hole (Table 2). A total of 274 adults of *S. adipata* (148 females, 126 males) emerged from these figs with no emergence of *Neosilba* spp. adults (Table 2). By contrast, a total of 27 adults of *S. adipata* (14 females, 13 males) and 42 adults of *Neosilba* spp. (22 females, 20 females) emerged from figs with one or more exit holes.

Of the 38 figs with holes that produced flies, 29 figs (76%) produced only *Neosilba* spp., 5 figs (13%) produced both *S. adipata* and *Neosilba* spp., and 4 figs (11%) produced *S. adipata* alone.

The mean number (±SE) of *S. adipata* adults (both sexes) that emerged from figs without a hole (6.9 ± 0.7 adults per fig) was significantly higher than observed for figs with the holes from which *Neosilba* spp. emerged (1.2 ± 0.1 adults per fig, both sexes) (Mann-Whitney, U = 109, *p* < 0.001). However, in co-infested figs, the mean number of *S. adipata* that emerged per fig was 3.0 ± 1.1 (both sexes), i.e., approximately half that observed when this species shared the fig with *Neosilba* spp.

The median period (18–19 days) over which *S. adipata* adults emerged from figs without an exit hole was similar for females and males (Log-rank test, *p* = 0.78) (Table 2). The median emergence period for *Neosilba* spp. was also similar for both sexes (Log rank test, *p* = 0.37) but was approximately one week longer (26–27 days) than observed for *S. adipata* (Log-rank test, *p* < 0.0001) (Table 2).

Interestingly, the median emergence time for *S. adipata* (both sexes) that emerged from figs with holes (co-infested by *Neosilba* spp.) was 17 days (95% C.I. 16–19 days, n = 27), which was a day shorter than observed in conspecifics (both sexes) that emerged from non-perforated figs (18 days, 95% C.I. 18–19 days, n = 274) (Log-rank test, *p* = 0.015). This suggests that figs were infested earlier by *S. adipata* or a reduced development time in the presence of *Neosilba* spp.

**Figure 4:**
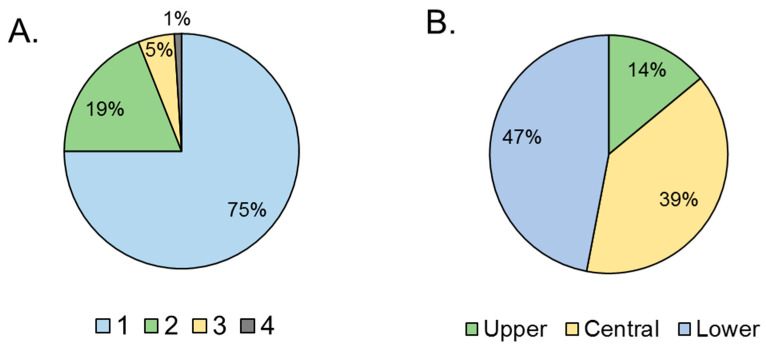
Damage associated with presence of *Neosilba* spp. (**A**) Percentage of figs with between 1 and 4 presumed larval exit holes per fig, and (**B**) location of presumed larval exit holes (from prior *S. adipata* infestation) in the lower, central or upper third of the figs. Percentages are based on a sample size of n = 130.

Figs infested by *Neosilba* spp. had an average (± SE) of 1.3 ± 0.05 holes per fig (n = 130). The frequency of holes varied significantly among figs (χ^2^ = 185; df = 3; *p* < 0.001). The majority of figs had just one hole (75%), with the remaining figs having two or three holes, and a single example with four holes (Figure 4A). The position of the holes also varied significantly (χ^2^ = 185; df = 2; *p* < 0.001). The holes were usually located in the central or lower third of the fig, with just 14% located in the upper third of the fig close to the pedunculus (Figure 4B).

#### 3.4.2. Samples Collected After the Harvest

The sample of figs without any exit holes collected after harvest in August was mainly infested by *S. adipata*. Of the 40 figs collected, *S. adipata* emerged from 35 (95%). The average number of *S. adipata* (8.4 ± 0.8 flies per fig; 49% males) was similar to that observed in figs collected before harvest (6.9 ± 0.7 flies per figs, Table 2) (Mann-Whitney U = 584, *p* = 0.218). Of particular interest was the fact that two individuals of *Neosilba* spp. emerged from two other figs (5%), which lacked exit holes—something that was not observed in figs collected prior to harvest.

The mean weight of figs collected after harvest (11.9 ± 0.5 g) was similar to that of figs collected before harvest (11.0 ± 0.3 g) (Welch’s t = 1.39, df= 52, *p* = 0.168) and the mean diameter of figs collected after harvest (2.7 ± 0.3 cm) was also similar to that observed in those collected before harvest (t = 0.078, df = 73, *p* = 0.938).

### 3.5. Crop Damage

**Figure 5:**
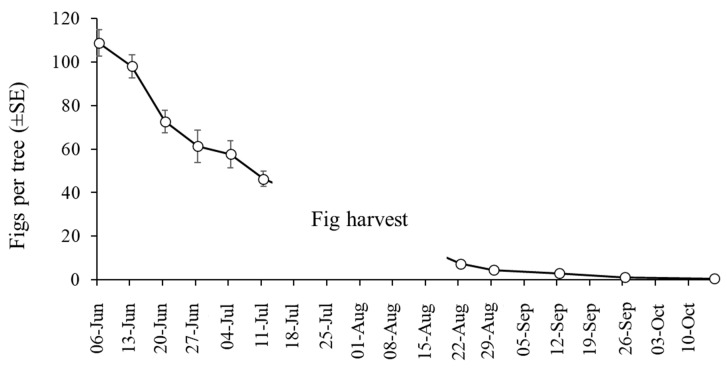
Mean (±SE) number of figs counted on 6 trees (pre-harvest) and 10 trees (post-harvest) that were randomly selected during sampling in 2024.

The mean number of figs per tree fell significantly during the course of the study (GLM: χ^2^ = 1401; df = 10; *p* < 0.001). About 65% of figs were lost in the month-long period between 6 June and 4 July due to lonchaeid infestation (Figure 5). Infested figs often fell from trees and were found on the ground, often in a rotten state (although fallen figs were not included in our collections). Few figs were present in the orchards after fig harvest in August and September, and sampling ceased in October 2024 due to a near absence of figs.

### 3.6. Species Identification and Phylogenetic Analyses

A sample of the specimens collected during this study was sent to the National Museum of Scotland for detailed examination of the male genitalia in order to accurately confirm identification to species level.

**Figure 6:**
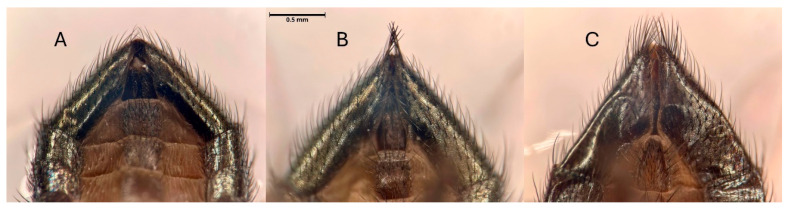
Apical segments of the abdomen (ventral view) of *Neosilba* spp. and based on genitalia examination considered as (**A**) *Neosilba batesi* (Curran, 1932) and *Neosilba recurva* sp. nov., (**B**) *Neosilba* sp.1 and (**C**) *Neosilba glaberrima*. Scale bar indicates 0.5 mm.

Individuals of *S. adipata* of both sexes were initially distinguished from *Neosilba* by distinctive characteristics such as their smaller body size, more metallic coloration, and intense red eye color, although species identity was confirmed using taxonomic keys [19,20]. Several species of *Neosilba* were initially separated based on external features on the apical segments of the male abdomen (Figure 6A–C), without a full dissection of the genitalia. Initial inspection of the apical segment morphology indicated the presence of *N. batesi* (Figure 6A), an unknown species *Neosilba* sp.1 with a small dense tuft of setae at the tip of the apical segment (Figure 6B), and *Neosilba glaberrima* (Wiedemann, 1830), with pronounced setae distributed around the tip of the apical segment (Figure 6C).

**Figure 7:**
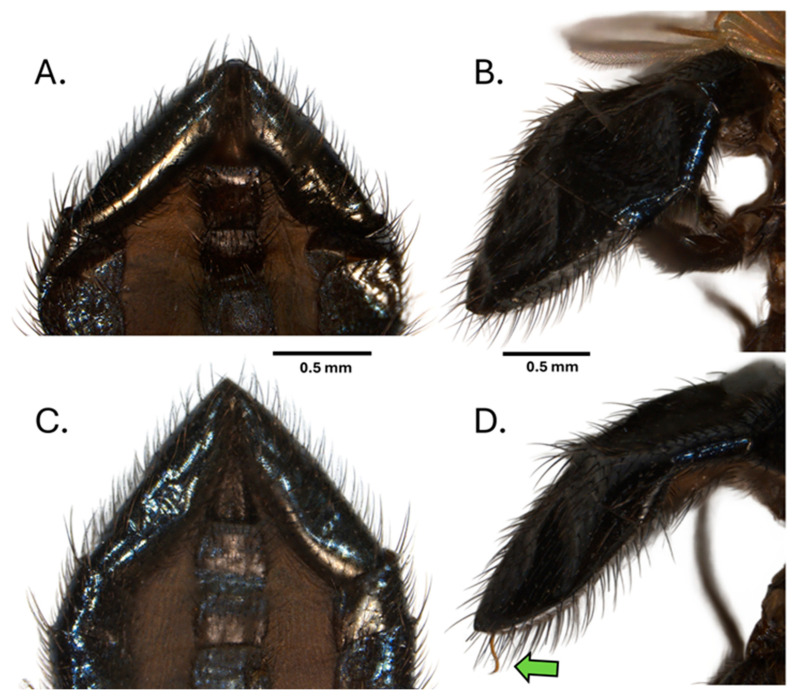
Apical segments of the abdomen of *Neosilba batesi*, (**A**) ventral view and (**B**) lateral view, and *Neosilba recurva* sp. nov. (**C**) ventral view and (**D**) lateral view. Scales bars indicate 0.5 mm. The green arrow in (**D**) indicates the curvature at the apex of the phallus, which may be visible externally in *N. recurva* when the specimens are adequately hydrated.

However, the dissection of several males that shared similar apical segment morphology to that of *N. batesi* revealed the presence of an additional previously undescribed species, *Neosilba recurva* sp. nov. Subsequent careful comparison of the apical segment morphology of *N. batesi* (Figure 7A,B) and *N. recurva* (Figure 7C,D) revealed that some minor differences were present but generally required observation of the unusual form of the phallus (Figure 7D).

The prevalence of males of the different *Neosilba* species varied during the study. Overall, based on the features outlined above, 64% of males corresponded to *N. batesi* and *N. recurva* (considered together due to their external similarities), 26% corresponded to *Neosilba* sp.1 and 10% to *N. glaberrima* (Table 3).

The specimens identified as *N. batesi* and *N. recurva* (considered together) were recorded emerging from figs from May to August, with just two males emerging in September and one in October. In contrast, *Neosilba* sp.1 emerged from figs collected from May to July, with just two individuals emerging from samples taken in August. Finally, *N. glaberrima* only emerged from samples collected in May, June and July (Table 3). None of the individuals corresponded to *N. flavitarsis*, a recently described species that was recorded from figs in October 2023 in the Tatatila municipality of Veracruz State [15].

**Figure 8:**
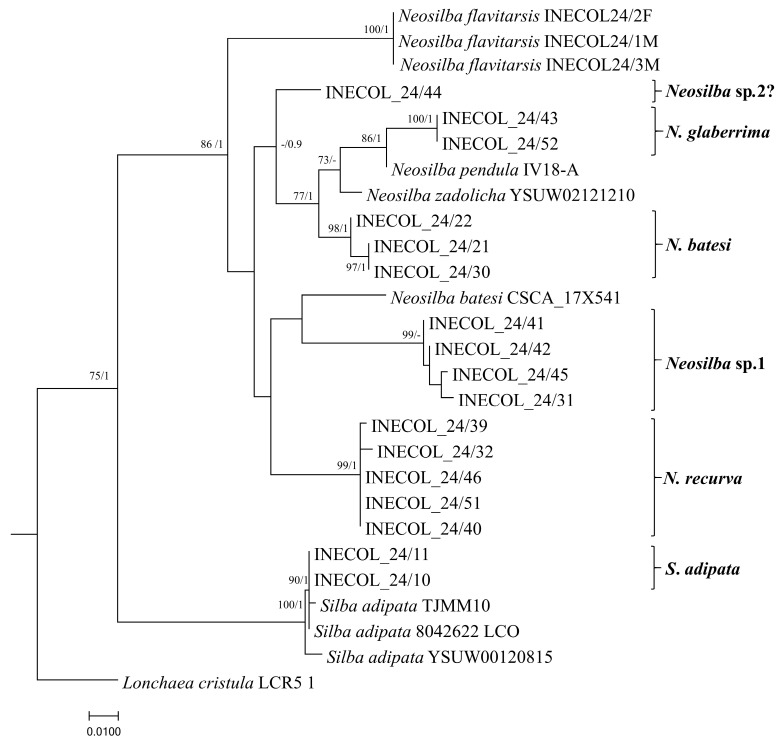
Maximum likelihood phylogenetic tree based on COI partial gene sequences illustrating the evolutionary relationships among lance flies (Diptera: Lonchaeidae). The tree was reconstructed using RAxML a GTR+G evolutionary model with 1000 rapid bootstrap replicates. The numbers at the nodes indicate Bootstrap values for 1000 replicates (BS ≥ 70%)/Bayesian Posterior Probabilities (BPP ≥ 0.9).

The COI gene region for each species was amplified by PCR and subjected to sequencing followed by phylogenetic analysis. Between one and five male individuals of each species were subjected to COI sequence analysis (Table 4). Specimens of *S. adipata* were confirmed to have close similarities with records present in the GenBank database. Both specimens of *S. adipata* from figs in Tatatila (INECOL_24/10 and INECOL_24/11) clustered together with sequences of conspecifics from Israel, Turkey, and Morelos State in Mexico (Figure 8). *Lonchaea cristula* McAlpine, 1964 was used as an outgroup for the phylogenetic tree. A female of this species was identified in torula traps in fig crops of Tatatila but not sequenced for this study.

Specimens identified as *N. batesi* were assigned to a clade that was not associated with the *N. batesi* sequence reported in Florida (CSCA-17X541) (Figure 8). *Neosilba recurva* and *Neosilba* sp.1 formed separate and well supported clusters that were clearly separated from the other species, confirming that these are two distinct species.

Specimens identified as *N. glaberrima* were grouped in a single clade clustered close to *N. pendula* (Bezzi, 1919): (IV18) from Brazil (Figure 8). A single individual (INECOL_24/44) that appeared to be *glaberrima*-like from the apical abdominal segment showed evident genetic differences from all the other species analyzed, suggesting that an additional species may be present, which is labeled as *Neosilba* sp.2? in Figure 8. However, the limited number of available specimens of this *glaberrima*-like group hindered a formal description. The characteristics of the male genitalia, particularly the shape of the phallus in one specimen from this group, showed some resemblance to *N. orbata* Galeano and Canal, 2021, described in Colombia [35]. Nonetheless, dissection of additional male specimens is required to clarify the identification. Finally, the recently recognized species from figs in Veracruz, *N. flavitarsis,* formed a separate branch from the other sequences reported in this study, supporting its status as a clearly distinct species. Importantly, the paucity of sequence data for *Neosilba* species in the GenBank (only four species with COI sequences from the 41 known species) resulted in lower than desirable confidence in some branches of the phylogenetic tree.

## 4. Discussion

This study documents an emerging lonchaeid pest complex that attacks fig production in the Tatatila region of Veracruz state in Mexico. Economic losses caused by lonchaeids in Veracruz are substantial, with a reduction of 65% in yield in the monitored area in the month prior to harvest. The damage caused by *S. adipata* to fig crops varies depending on fig cultivars, crop seasons, and regions but has been reported to reach up to 30–90% of fig production [6,17,38]. Indeed, the concern that these infestations have generated in the local population of fig growers of Tatatila brought this issue to our attention and prompted this initial study.

Despite their importance as pests in many parts of the world, studies on the biology and ecology of lonchaeids are relatively scarce. The first detection of figs infested by *S. adipata* was on 19 April, possibly from adults that emerged in late March. This aligns well with the initial detection of adults in the spring in the Mediterranean region [3]. In our samples, the number of *S. adipata* that emerged from each kilogram of figs increased markedly during the summer months (Table 1). This was reflected in an increase in the number of flies that developed in each fig, from 6–7 early in the season to typically 8–9 following the harvest, or exceptionally up to 22 flies from a single fig that was collected in August. Presumably, there are likely to be tradeoffs between the density of larvae per fig and the survival and fitness of the adults that emerge. Katsoyannos [3] reported that *S. adipata* lays between 1–4 eggs per fig in the spring and more than 50 eggs per fig in autumn. He proposed that this was due to differences in the fecundity of the spring and autumn generations. This idea merits further examination, although in our study, the reduced availability of figs following harvest also likely played a role in increasing the infestations of each fig. Based on the infestation of collected fig samples, we estimate that *S. adipata* has about five or six generations between March and October, which also reflects the annual number of generations in the Mediterranean region [3].

Infestation by lonchaeids was partially distinguishable by visual inspection of figs attached to trees. In *S. adipata*, the oviposition of a clutch of eggs beneath the scales of the ostiole [3] caused a red coloration normally associated with ripening that develops around the ostiole and which spreads quickly and often covers most of the fig (Figure 2A). In contrast, the emergence of *Neosilba* was observed (with the exception of two figs collected after fig harvest) in figs with an exit hole (Figure 2B). The holes are caused by *S. adipata* larvae leaving the fig to pupate in the soil. Exit holes have also been reported in green figs [7,38], although according to the observations of Drouet [17], when green unripe figs are infested by *S. adipata*, they turn purplish red if they belong to a dark-skinned variety (such as “Brown Turkey”), whereas varieties characterized by a light-skinned color at maturity remain entirely green. However, considerable variation in maturation patterns is observed in some figs presumably infested by *S. adipata* [17,38]. It is likely that in our study, *S. adipata* larvae had already left the figs to pupate, which would explain the absence of *S. adipata* adult emergence in most of the infested figs with an exit hole. In these cases, we assume that *Neosilba* spp. used the exit hole as an oviposition site to facilitate entry of the neonate larvae into the fig. Additionally, figs infested by *S. adipata* were smaller and with lower weight than those infested by *Neosilba* spp., suggesting that *S. adipata* attacks figs earlier than *Neosilba* spp. Unusually, Paniagua-Jasso et al. [6] reported observing occasional oviposition by *S. adipata* in the exit holes present in previously infested figs in central Mexico. We certainly never observed such behavior over the course of our study. Such observations require validation in future field studies as, to our knowledge, no other reports have documented this type of oviposition behavior for *S. adipata*. Although information related to the developmental duration of Lonchaeidae is scarce, *Neosilba* spp. adults required 14 additional days to emerge in comparison to *S. adipata*, which took 17–19 days (this study, “Brown Turkey” figs) or somewhat longer, 24–26 days, in “Black Mission” cultivar figs [6]. This difference could be explained if *Neosilba* spp. eggs were mostly laid following *S. adipata* infestation. However, this hypothesis did not find support from the oviposition behavior observed for some *Neosilba* females in the field (Figure 3B), in which some females were observed attempting to oviposit close to the equatorial region of the fig but not apparently in larval exit holes. Curiously, the mean number of holes observed on those figs (1.3 holes/fig) matched with the mean number of *Neosilba* spp. adults that emerged from figs (1.2 adults/fig), initially leading us to think that the holes were related to the oviposition of *Neosilba* spp. In any case, the oviposition behavior of the *Neosilba* flies remains uncertain and requires additional study to clarify the possible role of one or more of these species as primary pests of figs.

High infestations of *Neosilba* spp. were observed in figs at the end of May and early June (Table 1). *Neosilba* species have been reported as secondary invaders associated with primary invasion by tephritid flies in Mexico. The species involved include *N. certa* (Walker, 1853), *N. major* (Malloch, 1920), *N. oaxacana* McAlpine and Steyskal, 1982 and *N. peltae* McAlpine and Steyskal, 1982 [20]; however, no tephritids emerged from our fig samples. *Neosilba batesi* and *N. glaberrima* were identified as the most abundant members of the *Neosilba* species complex in Tatatila (Table 3). *Neosilba batesi* has been recorded in Guatemala, Mexico, Colombia, Panama, Florida (USA), Peru, Costa Rica, and El Salvador [9]. It is a polyphagous pest that has been reared from mango (*Mangifera indica* L.), sweet orange (*Citrus sinensis* L.), papaya (*Carica papaya* L.), peach palm (*Guilielma gasipaes* Kunth), and avocado (*Persea americana* Mill.) [20], as well as from various species in the genus *Annonas* [21]. *Neosilba batesi* has also been described as an emerging pest of avocado in Colombia [39] and recently in Mexico [40].

*Neosilba glaberrima* is also a polyphagous species that has been reared from figs within the Annonaceae, Anacardiaceae, Moraceae, Pasifloraceae, Solanaceae, Rutaceae, Myrtaceae, Malpighiaceae, and Oxalidaceae families [12,21,41,42]. This species was reared from figs in Brazil, together with other four additional *Neosilba* species: *N. bifida* Strikis and Prado, 2005, *N. certa*, *N. cornuphallus* Strikis, 2011, and *N. zadolicha* McAlpine and Steyskal, 1982 [12].

The analysis of the COI gene amplified sequences confirmed the identity of *S. adipata* but also indicated the presence of various species of *Neosilba* in addition to *N. flavitarsis*, which was detected in this region in 2023 [15]. Specimens identified as *N. batesi* formed a distinct separated cluster, different from the *N. batesi* sequence reported from Florida (CSCA-17X541), suggesting that the record from Florida requires confirmation (Figure 8). According to the characteristics of the apical segments of the abdomen, *N. batesi* was initially considered together with *N. recurva* when sorting through emerged adult material. These species could readily be differentiated by examination of the highly characteristic shape of the phallus of *N. recurva* (Figure 1B), and phylogenetic analysis strongly supported the species status of these individuals.

Sequences from *N. glaberrima* formed a specific cluster that appeared to be weakly related to *N. pendula* and *N. zadolicha* sequences reported from Brazil, but INECOL_24/43 and INECOL_24/52 represent the first sequences reported for *N. glaberrima* to our knowledge, so additional comparisons were not possible. The clear difference in the sequences of one specimen with a *glaberrima*-like abdomen morphology (Figure 6C) was also suggestive of the presence of an additional species (INECOL_24/44; labeled as *Neosilba* sp.2? in Figure 8). Both *Neosilba* sp.1 and *Neosilba* sp.2? require future clarification as to their species status once material becomes available. Although these sequences represent a three-fold increase in the number of *Neosilba* sequences registered in the GenBank database, the branches of phylogenetic tree are not well supported in some cases due to the paucity of available sequence information, which underlines the urgent need for extensive sequence studies from carefully identified specimens within the Lonchaeidae.

Fig growers in Tatatila previously reported only minor damage to crops from unidentified species of lance flies at the end of the cropping cycle and considered that such minor damage did not justify expenditure on chemical control measures. Recently, however, the movement of fig packaging boxes contaminated with larvae and pupae between growers in the states of Veracruz and Puebla has been identified as the likely source of the spread of *S. adipata* to Veracruz in 2022, although the origin of the *Neosilba* spp. populations in this region is unclear. Both *N. batesi* and *N. glaberrima* were previously recorded in Veracruz state, infesting several members of the pawpaw family, *Annonas* spp. (Annonaceae) [21]. The diversity of *Neosilba* in that region has never been studied, but the coexistence of *S. adipata* and *Neosilba* spp. has been reported in monitoring traps in fig orchards in central Mexico [43], even though fig infestation by *Neosilba* spp. was not reported by those authors.

## 5. Conclusions

The composition of lance fly species attacking figs in Veracruz, Mexico, varies throughout the production cycle, causing significant losses that necessitate the urgent adoption of integrated pest management strategies. Differences in oviposition behavior indicate that *Neosilba* spp. may act as secondary invaders, though some species might also be primary pests before harvest—an aspect that merits further study. Morphological and genetic analyses reveal considerable diversity within *Neosilba* spp., suggesting the presence of undescribed species. This study highlights the need for expanded molecular research on Lonchaeidae.

## Figures and Tables

**Table 1:** Number of females and males of *Silba adipata* and *Neosilba* spp. that that emerged from samples of figs of different weight that were collected during the crop season except for the harvest period.

Sample Date *	Weight of Figs (kg)	*Silba adipata*				*Neosilba* spp.			
		Females	Males	Total	Flies/kg	Females	Males	Total	Flies/kg
19 Apr 24	0.45	1	0	1	0.5	7	2	9	4.2
3 May 24	2.15	4	0	4	1.9	54	50	104	48.4
16 May 24	1.98	2	0	2	0.5	55	50	105	53.0
23 May 24	1.35	1	0	1	0.7	20	20	40	29.6
30 May 24	2.70	8	9	17	6.3	91	92	183	67.8
6 Jun 24	3.20	21	20	41	12.8	147	147	294	91.9
13 Jun 24	4.13	73	89	162	39.2	172	162	334	80.9
20 Jun 24	2.00	18	6	24	12.0	16	20	36	18.0
27 Jun 24	0.80	9	10	19	23.8	7	8	15	18.8
4 Jul 24	1.60	239	246	485	303.1	17	24	41	25.6
11 Jul 24	0.80	93	90	183	228.8	8	7	15	18.8
Harvest period									
22 Aug 24	1.14	257	228	485	425.4	34	29	63	55.3
29 Aug 24	0.53	60	93	153	288.7	5	10	15	28.3
12 Sep 24	0.129	17	22	39	302.3	1	0	1	7.8
27 Sep 24	0.123	3	3	6	48.8	1	2	3	23.1
15 Oct 24	0.101	16	10	26	257.4	0	1	1	9.9

* No flies emerged from fig samples collected in the period 13 March–12 April 2024.

**Table 2:** Characteristics of infested figs and emergence of flies from samples of 40 figs with or without holes.

Fig Characteristics				No. Flies/Fig (±SE)		Emergence Time (Days) (95% C.I.)	
	Fig Weight (g) (±SE) ^1^	Fig Diameter (cm) (±SE) ^1^	Species Emergence from Figs (%) ^2^	Females	Males	Females	Males
Figs without hole	11.0 ± 0.3 a	2.7 ± 0.1 a	*S. adipata* 100%	3.7 ± 0.5	3.2 ± 0.4	19 (18–20)	18 (18–21)
			*Neosilba* spp. 0%	-	-	-	-
Figs with hole	13.8 ± 0.5 b	3.0 ± 0.1 b	*S. adipata* 24%	1.6 ± 0.4	1.6 ± 0.5	17 (15–21)	17 (16–21)
			*Neosilba* spp. 89%	0.7 ± 0.2	0.6 ± 0.1	27 (26–34)	26 (26–28)

^1^ Values followed by different letters differ significantly (*t*-test, *p* < 0.05). ^2^ Indicates the percentage of figs from which each genus emerged.

**Table 3:** Emergence by month of different species of *Neosilba* males that were identified morphologically as *N. batesi* and *N. recurva* (considered together), *Neosilba* sp.1 or *N. glaberrima*.

	Species Composition (%)			
Month	*N. batesi* and *N. recurva*	*Neosilba* sp.1	*N. glaberrima*	Total Number of Males Identified
May	72%	20%	8%	66
June	57%	30%	11%	95
July	52%	35%	13%	113
August	95%	5%	0	39
September	100%	0	0	2
October	100%	0	0	1
Total:	64% (n = 202)	26% (n = 82)	10% (n = 32)	316

**Table 4:** Species, specimen voucher code, collection site, and GenBank accession numbers for lonchaeid species used in the construction of the phylogenetic tree. Adult emergence date was included for the specimens collected in Veracruz State, Mexico that were evaluated in this study.

Species (Emergence Date)	Voucher Code	State, Country	GenBank Number
*Neosilba batesi* (23/05/24)	INECOL_24/21	Veracruz, Mexico	PV504763
*Neosilba batesi* (23/05/24)	INECOL_24/22	Veracruz, Mexico	PV504764
*Neosilba batesi* (02/07/24)	INECOL_24/30	Veracruz, Mexico	PV504765
*Neosilba batesi*	CSCA_17X541	Florida, USA	MW283302
*Neosilba glaberrima* (27/06/24)	INECOL_24/43	Veracruz, Mexico	PV504766
*Neosilba glaberrima* (23/05/24)	INECOL_24/52	Veracruz, Mexico	PV504767
*Neosilba recurva* (02/07/24)	INECOL_24/32	Veracruz, Mexico	PV522074
*Neosilba recurva* (27/06/24)	INECOL_24/39	Veracruz, Mexico	PV522075
*Neosilba recurva* (16/09/24)	INECOL_24/40	Veracruz, Mexico	PV522076
*Neosilba recurva* (13/09/24)	INECOL_24/46	Veracruz, Mexico	PV522077
*Neosilba recurva* (23/05/24)	INECOL_24/51	Veracruz, Mexico	PV522078
*Neosilba* sp.1 (02/07/24)	INECOL_24/31	Veracruz, Mexico	PV504768
*Neosilba* sp.1 (02/07/24)	INECOL_24/41	Veracruz, Mexico	PV504769
*Neosilba* sp.1 (17/07/24)	INECOL_24/42	Veracruz, Mexico	PV504770
*Neosilba* sp.1 (15/08/24)	INECOL_24/45	Veracruz, Mexico	PV504771
*Neosilba* sp.2? (17/07/24)	INECOL_24/44	Veracruz, Mexico	PV504772
*Neosilba flavitarsis*	INECOL24/1M	Veracruz, Mexico	PQ834830
*Neosilba flavitarsis*	INECOL24/2F	Veracruz, Mexico	PQ834831
*Neosilba flavitarsis*	INECOL24/3M	Veracruz, Mexico	PQ834832
*Neosilba pendula*	IV18-A	N/D *	OQ160349
*Neosilba zadolicha*	YSUW02121210	Brazil	KR262649
*Silba adipata* (03/05/24)	INECOL_24/10	Veracruz, Mexico	PV504773
*Silba adipata* (03/05/24)	INECOL_24/11	Veracruz, Mexico	PV504774
*Silba adipata*	YSUW00120815	Israel	KR262672
*Silba adipata*	TJMM10	Morelos, Mexico	OM949837
*Silba adipata*	8042622-LCO	Turkey	MK450119
*Lonchaea cristula*	LCR5-1	Colombia	MZ189773

* N/D—not determined, probably Brazil.

## Data Availability

The original contributions presented in this study are included in the article and Supplementary Material. Further inquiries can be directed to the corresponding authors.

## Supplementary Materials

The following supporting information can be downloaded at: https://www.mdpi.com/article/10.3390/insects16050458/s1, Figure S1: Lateral view of paratype adult male of *Neosilba recurva* sp. nov. Table S1: Mean and median emergence time in days for females and males of *Silba adipata* and *Neosilba* spp. along the different dates of fig collection in 2024.
