# Supplementary material for: A Complex of Lance Flies (Diptera: Lonchaeidae) Infesting Figs in Veracruz, Mexico, with the Description of a New Species
Source: Insects. 2025 Apr 27;16(5):458. doi: 10.3390/insects16050458 (PMC12112198; doi:10.3390/insects16050458)
Supplement: Supplementary file 1 [file insects-16-00458-s001.zip › insects-3616177-supplementary Table S1.pdf]

Table S1. Mean and median emergence time in days for females and males of *Silba adipata* and *Neosilba* spp. along the different dates of fig collection in 2024.

	Silba adipata		***Neosilba* spp.**	
**Week**	**Females**	**Males**	**Females**	**Males**
19-Apr-24	17	-	30.8	30
3-May-24	13	13	21.6	23.2
16-May-24	14	14	19.4	20.1
23-May-24	12	-	25.9	24.6
30-May-24	12.4	12.8	24.9	25.6
6-Jun-24	16.7	15.4	30.1	30.3
13-Jun-24	16.5	15	30.5	31.2
20-Jun-24	27.4	23.4	35.9	37
27-Jun-24	24.4	23.5	30.4	29
4-Jul-24	17.3	17	34	32.7
11-Jul-24	18.3	17.3	30.7	33
Harvest period				
22-Aug-24	21.8	19	29.9	29.2
29-Aug-24	17.9	18.6	38.6	36.4
12-Sep-24	18.6	16	32	-
27-Sep-24	13.6	14.3	31	33.5
15-Oct-24	20.9	22.4	-	30
**Mean**	**17.6**	**17.3**	**29.7**	**29.7**
**Median**	**17.1**	**16.5**	**30.5**	**30**
**95% C.I.**	**14-21.8**	**15-23.4**	**29.9-34**	**29-33.5**
