# Supplementary figures and images for: A Complex of Lance Flies (Diptera: Lonchaeidae) Infesting Figs in Veracruz, Mexico, with the Description of a New Species
Source: Insects. 2025 Apr 27;16(5):458. doi: 10.3390/insects16050458 (PMC12112198; doi:10.3390/insects16050458)

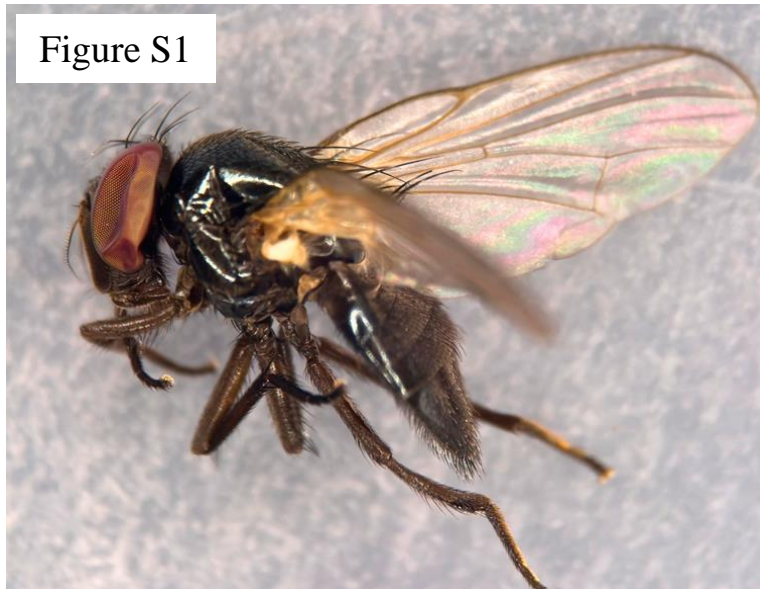

Figure S1. Lateral view of paratype adult male of *Neosilba recurva* sp. nov.

Supplement: Supplementary file 1 [file insects-16-00458-s001.zip › insects-3616177-supplementary Figure S1.pdf]
